# Supplementary material for: WordCommentsAnalyzer: A windows software tool for qualitative research
Source: F1000Res. 2018 Sep 4;7:536. Originally published 2018 May 3. [Version 2] doi: 10.12688/f1000research.14819.2 (PMC6137411; doi:10.12688/f1000research.14819.2)
Supplement: Supplementary file 3 [file f1000research-7-17614-s0002.tgz › b2a79496-4ada-47c4-9682-a99ce867836d.zip!/Eight Iranian Health Journals/Salmand_Iranian Journal of Ageing _44.docx]

© Salmand_Iranian Journal of Ageing . This work is licensed under CC BY-NC 4.0 (https://creativecommons.org/licenses/by-nc/4.0/).

Volume & Issue: دوره 2 - شماره 2

Title: مشخصات تشخیصی و درمانی سردرد در سالمندان (مقاله مروری)

http://salmandj.uswr.ac.ir/article-1-1-fa.html

از نظر بالینی تفاوتی بین ویژگی‌های انواع سردرد در سالمندان و سایر افراد وجود ندارد. ولی تشخیص افتراقی اورای میگرنی از حملات گذرای ایسکمی مغزی سالمندان که معمولا ریسک فاکتورهای عروقی را نیز دارند مشکل است. سالمندان کمتر از جوانان دچار سردرد می‌شوند. سردرد تنشی مزمن، شایع‌ترین سردرد اولیه در سالمندان است. درد گاه‌گاهی نیمه سر و سردرد ناشی از آرتریت سلول ژیانت ویژه سالمندان است. سردردهای ثانویه مانند سردرد ناشی از اسپوندیلوز گردنی و تومورهای مغزی در سالمندان شایعتر از جوانان است. سالمندان داروهای سردرد مانند ارگوتامین و تریپتان‌ها و تری‌سیکلیک‌ها را به‌خوبی تحمل نمی‌کنند. نورالژی تری ژمینال اغلب در سالمندان دیده می‌شود و درمان دارویی آن در افراد مسن مشکل است. سردرد می‌تواند تظاهر اصلی افسردگی در سالمندان باشد. سردردهای ناشی از اختلالات طب داخلی مانند هیپرتانسیون و بیماری‌های انسدادی مزمن ریه در افراد پیر اهمیت دارند. در هر سالمند با سردرد ناگهانی و بسیار شدید باید به فکر خونریزی ساب آراکنوئید باشیم. به‌ویژه اگر با کاهش هوشیاری و ردور گردن همراه باشد. افراد پیر به علت ابتلا به بیماری‌های متعدد کلکسیونی از داروها را مصرف نموده و سردردهای ناشی از مصرف دارو در آن‌ها شایع است. در بیمار سالمندی که بدون سابقه قبلی دچار سردرد شدید شده و اختلالات طبی و مصرف داروی مسبب آن را ذکر نمی‌کند برای بررسی ضایعات ساختمانی داخل جمجمه باید تصویربرداری از مغز انجام شود. عده‌ای از سالمندان هم‌پوشانی چند نوع از سردرد را دارند.

Title: بررسی اثر Romberg Exercise بر میزان زمین‌خوردن سالمندان با سابقه زمین‌خوردن (مقاله پژوهشی)

http://salmandj.uswr.ac.ir/article-1-2-fa.html

اهداف: آیا تمرین رومبرگ می تواند خطر زمین خوردن را کاهش دهد؟ این روش دو موقعیت مهم یعنی حفظ بالانس در شرایط کاهش سطح اتکا و کاهش اطلاعات بینایی که دو عامل مهم زمین‌خوردن سالمندان هستند را هدف قرار داده است و به‌طور وسیعی توسط دیگر محققان به عنوان یک روش ارزیابی بالانس به‌کار رفته است اما به‌عنوان روش درمان استفاده نشده است. مواد و روش‌ها: این تحقیق به روش RCT انجام شده است. 70 نفر از سالمندانی که دارای سن 65 سال یا بیشتر بوده و در شش ماه گذشته دو بار یا بیشتر زمین خورده باشند انتخاب شده و سپس آن‌ها را به صورت تصادفی به دو گروه شاهد و مداخله تقسیم نمودیم. سپس گروه مداخله به مدت سه ماه یک روز در میان، هر روز به مدت 45 دقیقه تمرین رومبرگ داده شدند و پس از اتمام دوره سه ماهه هر دو گروه به مدت 6 ماه از نظر تعداد زمین‌خوردن، میزان و شدت عوارض و شکستگی تحت نظر قرار گرفتند. یافته‌ها: در تعداد زمین خوردن، در افرادی که پیگیری کامل شدند، بین دو گروه تفاوت معناداری مشاهده نشد (0/90=P). درصد افرادی که زمین خوردند نیز تفاوت معناداری بین دو گروه شاهد و مداخله نشان نداد [%52/4 درمقابل %50 (P=0/87)]. در بررسی ارتباط میزان اثربخشی تمرینات با سن، نتایج به‌دست آمده ارتباط معناداری را نشان ندادند. (%12= power،0/46=P) در آنالیز Univariate analysis of variance انجام شده، تعداد داروهای مصرفی سالمندان، با میزان اثربخشی تمرینات در کاهش سقوط، ارتباط معناداری داشت. (0/460=P). نتیجه‌گیری: اگرچه تست‌های آماری نتوانستند تفاوت معناداری را نشان دهند، این ناتوانی را میتوان ناشی از کم بودن قدرت این مطالعه (power) برای پیدا کردن این ارتباط دانست، لذا برای دستیابی به نتیجه قطعی‌تر در این باره، به مطالعاتی با حجم نمونه بیشتر نیاز است. از طرفی سقوط، وابسته به چندین عامل است، کارآزمایی‌های بالینی اخیر ثابت کرده است که دخالت‌های چندمنظوره، بهترین نتیجه را در کاهش سقوط نشان می دهد.

Title: الگوی هزینه و بیماری در خدمات بستری سالمندان تحت پوشش سازمان بیمه خدمات درمانی شهرستان دامغان طی سال 1384

http://salmandj.uswr.ac.ir/article-1-3-fa.html

اهداف: بالارفتن امید به زندگی و افزایش جمعیت سالمند علی‌رغم بازتاب رضایت بخش خود، مسائل و مشکلاتی را در سال‌های پایانی عمر، به‌ویژه در رابطه با سلامت و بیماری به همراه دارد. بیماری‌های مزمن به لحاظ افزایش هزینه های درمان و نیاز به مراقبت‌های طولانی از آن جمله اند. مواد و روش‌ها: در بحث‌های مربوط به اپیدمیولوژیک ترنزیشن بطور کلی به جایگزینی بیماری‌های مزمن با بیماری‌های عفونی اشاره می‌شود، اما مصادیق آن به تفضیل مورد بحث و گفت‌وگو قرار نمی‌گیرد. تحلیل دقیق‌تر این موضوع که کدام بیماری مزمن با کدام ویژگی‌های جمعیتی، اقتصادی - اجتماعی و خدماتی اولویت را در سلسه مراتب تولید و مصرف خدمات به خود اختصاص می‌دهد نیازمند مطالعات موردی است تا نشانه‌ها و الگوهای ترنزیشن را در هر جامعه‌ای به‌طور برجسته‌ای بتوان معرفی نمود، این مطالعه تا حد بسیار ناظر به این موضوع است. یافته‌ها: مطالعه حاضر در دو بیمارستان شهرستان دامغان طراحی و اجرا شده است و طی آن پرونده کلیه سالمندان بیمار (بالای 60 سال) بستری تحت پوشش بیمه خدمات درمانی در جست‌وجوی یافتن الگوهای مسلط بیماری و هزینه‌های درمان مورد بررسی و تحلیل قرار گرفته اند. نتیجه‌گیری: عمده‌ترین دلیل بستری، ابتلا به بیماری‌های دستگاه گردش خون و بیشترین جراحی انجام شده، در رابطه با جراحی کاتاراکت گزارش شده است. بیماری‌های اعصاب و روان بالاترین میانگین هر بار بستری را به خود اختصاص داده است.

Title: بررسی انواع اختلالات خواب و روش‌های مقابله با آن‌ها در سالمندان (مقاله پژوهشی)

http://salmandj.uswr.ac.ir/article-1-4-fa.html

اهداف: خواب یکی از نیازهای اساسی انسان است و هرگونه اختلال در جریان طبیعی آن علاوه بر ایجاد مشکلات روانی می تواند کارآیی فرد را نیز کاهش دهد. اگرچه اختلالات خواب در هر سنی رخ می دهد، افراد پیر غالبا با مشکلات زیادی جهت دستیابی به یک خواب راحت مواجه هستند. بر این اساس هدف این مطالعه تعیین انواع اختلالات خواب و روش های مقابله با آنها در سالمندان می باشد. مواد و روش‌ها: نوع مطالعه توصیفی بوده که با مراجعه با منازل سالمندان ضمن مصاحبه با آن‌ها، پرسش‌نامه ای که از قبل در ارتباط با اختلالات خواب (دیس سومنیا، پاراسومنیا) و روش های مقابله با آن‌ها (رفتاری، شناختی، رعایت بهداشت خواب و دارو درمانی) تهیه شده بود تکمیل گردید. یافته‌ها: نتایج نشان داد که شیوع دیس سومنیا در سالمندان 67درصد بود و در 61درصد آنها بیخوابی وجود داشت، که بیشتر (28/5درصد) به صورت بی خوابی (اولیه، متناوب و انتهایی) بود. شیوع پاراسومنیا 29درصد و بیشتر (14درصد) به فرم دیدن خواب های ترسناک بود. در رابطه با استفاده از روش های مقابله، 57درصد از روش های رفتار درمانی استفاده می کردند که بیشتر (25درصد) به صورت تمرکز بر اندام ها قبل از خواب بود، 95/5درصد روشهای شناختی را درک کرده اند که بیشتر (26درصد) به صورت درک تاثیر سن بر خواب بود. 100درصد نسبت به موارد بهداشت خواب آگاه بودند و بیشتر نمونه ها (39درصد) حداقل با 4 مورد از موارد بهداشتی موثر بر خواب آشنا بودند و 20درصد از دارو استفاده میکردند. نتیجه‌گیری: یافته های فوق نشان می دهد که اختلالات خواب در سالمندان شایع بوده و با توجه به تاثیر مثبت روش‌های مختلف در مقابله با اختلالات خواب، تاکید بر آموزش این روش‌ها می‌تواند به طور مطلوب مشکلات موجود در اختلالات خواب را بر طرف سازد.

Title: بررسی عوامل مؤثر بر موقعیت سالمند در خانواده (مقاله پژوهشی)

http://salmandj.uswr.ac.ir/article-1-5-fa.html

اهداف: هدف از این مطالعه، بررسی علل و عوامل موثر بر موقعیت سالمند و خانواده بوده است. این بررسی سعی دارد با شناخت عوامل تثبیت‌کننده و بهبوددهنده وضعیت و موقعیت فرد سالمند در خانواده گامی هر چند کوچک در جهت حفظ و تعالی این جایگاه و موقعیت برداشته و کمکی هر چند اندک در بهبود و ارتقا شرایط زیست سالمندان در جمع خانوادگی بنماید. مواد و روش‌ها: پژوهش حاضر، به روش پیمایشی و با استفاده از تکنیک مصاحبه و پرسش‌نامه در یک جامعه آماری متشکل از سالمندان بالای 65 سال مقیم در خانواده در منطقه 7 تهران و در یک جمعیت نمونه 200 نفری از این جامعه آماری (به روش تصادفی ساده) انجام یافته است. یافته‌ها: پس از تجزیه و تحلیل داده‌ها مشخص گردید که میان کلیه متغیرهای مستقل مطروحه در این بررسی و متغیر وابسته موقعیت سالمند در خانواده رابطه معنی‌داری وجود دارد و با تاکید بر دو متغیر مستقل گستردگی خانواده و مذهبی بودن خانواده مشخص گردید که این دو متغیر از تاثیرگذاری بالاتری برخوردار هستند. طبق نتایج حاصل از رگرسیون چندمتغیره، دو متغیر مستقل مذکور با متغیر وابسته مشخص شد که مجموعا 47.2درصد از تغییرات متغیر وابسته توسط این دو متغیر مستقل پوشش داده می‌شوند که در این میان متغیر مستقل گستردگی خانواده از سهم بیشتری برخوردار است. نتیجه‌گیری: یافته‌های تحقیق حاضر نشان می‌دهد که فاکتورها و عوامل مختلفی مانند سن، میزان تحصیلات، وضعیت سلامتی، میزان همکاری، وضعیت اقتصادی، نوع خانواده و بالاخره میزان مذهبی‌بودن خانواده فرد سالمند بر موقعیت افراد سالمند در خانواده اثر گذارند که در این میان نوع خانواده از درجه اهمیت بالاتری برخوردار است. لذا با شناخت و مطالعه پیرامون این عوامل و انجام اقدامات و برنامه‌ریزی‌های مناسب و موثر می توان ضمن پیشگیری و کاهش مشکلات فعلی و آتی سالمندان، موجبات ارتقا و بهبود هرچه بیشتر موقعیت آنان را در خانواده فراهم آورد.

Title: مقایسه وضعیت سالمندان مقیم آسایشگاه و مقیم خانواده در ایران (مقاله پژوهشی)

http://salmandj.uswr.ac.ir/article-1-6-fa.html

اهداف: رشد صعودی جمعیت سالمندان کشورمان و توجه جامعه به پدیده سالمندی، موجبی شده تا در راستای ضرورت مطالعه و شناخت ابعاد مختلف مسایل و مشکلات جمعیت سالمندی، به مقایسه وضعیت سالمندان مقیم آسایشگاه و مقیم در خانواده بپردازیم. مواد و روش‌ها: این مطالعه از نوع توصیفی مقطعی (Cross sectional) است. در این تحقیق 504 نفر سالمند بالای 60 سال، مورد بررسی قرار گرفتند، 246 نفر سالمندان مقیم آسایشگاه های سالمندی سراسر کشور به‌عنوان گروه مقیم آسایشگاه و 258 نفر سالمندان غیر آسایشگاهی، به‌عنوان گروه مقیم خانواده انتخاب شدند. نمونه گیری به روش تصادفی سیستماتیک انجام شده است. ابزار جمع آوری داده ها، عبارت است از: پرسش نامه ای که توسط محقق تدوین شده و بوسیله مصاحبه با سالمند، خانواده و مشاهدات محقق تکمیل گردیده است. یافته‌ها: میانگین سنی سالمندان مقیم آسایشگاه 75/4 سال و سالمندان مقیم خانواده 71/3 سال بوده است. زنان بیش از مردان روانه آسایشگاه می شوند. پایگاه اجتماعی و اعتقادات مذهبی در میان سالمندان مقیم خانواده بالاتر بوده است. نداشتن فرزند، بی همسری، نداشتن خانواده، تنهایی و بی کسی، نداشتن مسکن و نگرش منفی اعضا خانواده به سالمندی در میان سالمندان مقیم آسایشگاه بیشتر است. نتیجه‌گیری: با توجه به نتایج بدست آمده، به‌نظر می رسد، افول پایگاه اجتماعی سالمندان، بی‌همسری، نداشتن خانواده، بی‌مسکنی و نگرش منفی اعضا خانواده نسبت به فرد سالمند از عوامل موثر بر اقامت سالمندان در آسایشگاه‌های سالمندی است که برنامه‌ریزان و مسئولان اجرایی امور اجتماعی و گروه سالمندی باید به آن توجه داشته باشند.

Title: بررسی مقایسه ای یادگیری ضمنی توالی حرکتی در سالمندان و جوانان

http://salmandj.uswr.ac.ir/article-1-1235-fa.html

هدف: توالی حرکتی اساس بسیاری از رفتارهای هوشیارانه انسان است، چرا که رفتارهای پیچیده حرکتی زنجیره ای از رفتارهای اولیه است. سالمندی شناختی بخشی از فرایند سالمندی است که موجب کاهش توانایی های شناختی سالمندان از جمله یادگیری می گردد. هدف این پژوهش مقایسه یادگیری ضمنی در سالمندان و جوانان است. روش بررسی: ابتدا نرم افزار تخصصی ارایه محرک های متوالی و ثبت زمان و خطای پاسخ طراحی گردید. این نرم افزار در تکلیف یادگیری حرکتی ارایه شده زمان پاسخ و تعداد خطا را ثبت می نمود. در این پژوهش دو گروه سالمند و جوان (هر گروه 15 نفر) در تکلیف یادگیری ضمنی شرکت داده شدند. تکلیف حرکتی ارایه شده به نحوی بود که در مراحل اول و ششم محرک ها به صورت نامنظم و در سایر مراحل به صورت منظم ظاهر می شدند. یافته ها: آزمون تحلیل واریانس برای اندازه گیری های مکرر زمان پاسخ و خطای پاسخ، تی زوج شده برای مقایسه داده های منظم و نامنظم یک گروه و تی مستقل برای مقایسه داده های دو گروه سالمند و جوان مورد استفاده قرار گرفت. نتایج نشان داد که زمان پاسخ در توالی های منظم و نامنظم در گروه جوانان کاهش می یابد ولیکن در گروه سالمندان فقط در توالی منظم کاهش می یابد. و تعداد خطا و سرعت پاسخ در سالمندان کمتر از جوانان بود. نتیجه گیری: بر اساس یافته های پژوهش حاضر در سالمندان یادگیری ضمنی توالی حرکتی اتفاق می افتد. و دقت یادگیری ضمنی سالمندان از جوانان بیشتر است. همچنین یادگیری مهارت حرکتی به صورت ضمنی برای آموزش مهارت های حرکتی به سالمندان، خاصه مهارتهای نیازمند دقت، روش مناسبی می باشد.

Title: سالمندی موفق در پرتو مذهب و باورهای دینی (مقاله مروری)

http://salmandj.uswr.ac.ir/article-1-7-fa.html

هدف از مقاله حاضر، بررسی مروری رابطه مذهب و باورهای دینی با سالمندی موفق (سلامت روان) است. افسردگی و اضطراب در بین سالمندان شایع است. واژه سالمندی موفق توسط بیلتزو بیلتز (1990) پیشنهاد شد. تعاریف متعددی از سالمندی موفق و مذهب بیان گردیده است. نقش مذهب و باورهای دینی در زمینه سالمندی موفق بررسی شده و تحقیقات متعددی در این رابطه انجام شده است. آلپورت دو نوع جهت‌گیری مذهبی درونی و بیرونی را مطرح کرده که تحقیقاتی هم در این زمینه صورت گرفته است. نتایج نشان می دهد که بین سالمندی موفق و جهت‌گیری مذهبی رابطه وجود دارد. و نیز جهت‌گیری مذهبی بیرونی با اختلال در سلامت روان و افسردگی و جهت‌گیری درونی با سلامت روان رابطه دارند و نیز اختلال روانی و افسردگی در بین سالمندان مقیم خانه سالمندان بیشتر از سالمندان مقیم جامعه است. ضمن آنکه سالمندانی که مقیم مرکز نگهداری سالمندان بودند جهت‌گیری مذهبی بیرونی داشتند. تحقیقات نشان می دهد که شدت افسردگی سالمندان با میزان نگرش و عملکرد دینی و معنوی آن ها بالاتر بود، افسردگی کمتری داشتند.

Title: آینده سالمندی و هزینه‌های خدمات سلامت: هشداری برای نظام سلامت کشور (مقاله مروری)

http://salmandj.uswr.ac.ir/article-1-8-fa.html

این مقاله به موضوع افزایش جمعیت سالمندی، از منظر هزینه‌های بخش سلامت طی دهه‌های آینده می‌پردازد. در ابتدا به دموگرافی سالمندان در جهان و ایران اشاره شده و سپس موضوع هزینه‌های خدمات سلامت در دوران سالمندی در مقایسه با سایر گروه‌های سنی مورد بحث قرار می گیرد. با قبول این واقعیت که هزینه‌های بهداشت و درمان در دوران سالمندی چندین برابر سایر سنین است، اهمیت محاسبه و پیش‌بینی این هزینه‌ها بیان شده و روش برآورد و پیش‌بینی آن شرح داده می‌شود. در بحث بر این واقعیت تاکید شده است که ایران نیز مانند سایر کشورها طی دهه‌های آینده با افزایش جمعیت سالمند مواجه خواهد بود و این امر، بر اساس تخمین انجام شده و به شرط ثابت‌بودن سایر متغیرها، نیازمند افزایش هزینه سرانه سلامت تا سال 2050 میلادی به میزان 5، 2 برابر است. در پایان، اهمیت توجه به موج سالمندی در چند دهه آینده به سیاست‌گذاران و تصمیم‌گیران عرصه سلامت کشور، توصیه شده است.
